# Exploring genetic architecture of grain yield and quality traits in a 16-way *indica* by *japonica* rice MAGIC global population

**DOI:** 10.1038/s41598-019-55357-7

**Published:** 2019-12-20

**Authors:** Hein Zaw, Chitra Raghavan, Arnel Pocsedio, B. P. Mallikarjuna Swamy, Mona Liza Jubay, Rakesh Kumar Singh, Justine Bonifacio, Ramil Mauleon, Jose E. Hernandez, Merlyn S. Mendioro, Glenn B. Gregorio, Hei Leung

**Affiliations:** 1 Plant Breeding Division, International Rice Research Institute (IRRI), DAPO Box, 7777 Metro, Manila Philippines; 2 Institute of Crop Science, University of the Philippines Los Baños (UPLB), Laguna, 4031 Philippines; 3 Plant Biotechnology Center, Department of Agriculture, Shwe Nanthar, Mingaladon Tsp, Yangon, Myanmar; 4 International Center for Biosaline Agriculture, Dubai, United Arab Emirates

**Keywords:** Genetics, Plant sciences

## Abstract

Identification of Quantitative Trait Loci (QTL) has been a challenge for complex traits due to the use of populations with narrow genetic base. Most of QTL mapping studies were carried out from crosses made within the subspecies, either *indica* × *indica* or *japonica* × *japonica*. In this study we report advantages of using Multi-parent Advanced Generation Inter-Crosses global population, derived from a combination of eight *indica* and eight *japonica* elite parents, in QTL discovery for yield and grain quality traits. Genome-wide association study and interval mapping identified 38 and 34 QTLs whereas Bayesian networking detected 60 QTLs with 22 marker-marker associations, 32 trait-trait associations and 65 marker-trait associations. Notably, nine known QTLs/genes *qPH*_*1*_/*OsGA20ox2*, *qDF*_*3*_/*OsMADS50*, *PL*, *QDg1*, *qGW-5b*, *grb7-2*, *qGL*_*3*_/*GS*3, *Amy6*/*Wx* gene and *OsNAS3* were consistently identified by all approaches for nine traits whereas *qDF*_*3*_/*OsMADS50* was co-located for both yield and days-to-flowering traits on chromosome 3. Moreover, we identified a number of candidate QTLs in either one or two analyses but further validations will be needed. The results indicate that this new population has enabled identifications of significant QTLs and interactions for 16 traits through multiple approaches. Pyramided recombinant inbred lines provide a valuable source for integration into future breeding programs.

## Introduction

Rice is a major food crop for over half of the world population, accounting for almost 90% of production of global rice by Asian countries^1^. With the increase of world population, rice production has to be doubled by 2050^2^. The production of rice has been significantly improved after the development of semi-dwarf cultivars and hybrid rice^3^. However, in the last decades, rice yield was not significantly improved and reached into a projected rice production^4^. To ensure food security, declining in genetic gain, narrow genetic base of the modern rice varieties, biotic and abiotic stress pressure, increasing demand for more quantity and better quality of rice are some of the concerns for rice breeders^2,3,5^. In practice, most of the economically important traits display complex genetic architecture that are under polygenic control and often influenced by extensive genotype × environment (G×E) interactions.

Breeders and geneticists traditionally used bi-parental populations for Quantitative Trait Loci (QTL) mapping and varietal development. A number of mapping studies have been carried out in bi-parental populations for detecting QTLs for grain yield and quality traits because of the ease of population development and availability of a wide range of statistical analysis tools^6–13^. Bi-parental populations such as Recombinant Inbred Lines (RILs), Backcross Inbred Lines (BILs), Near Isogenic Lines (NILs), Advanced Inter-Cross (AIC) and Double Haploid (DH) have been found to be effective in mapping of large-effect QTLs^14–20^. The weakness of the bi-parental population is that loci are mapped with low mapping resolution as a result of the limited recombination^21^. Additional mapping is still required to fine map the QTLs with small effects. In contrast association mapping exploits linkage disequilibrium (LD) to localize small and large-effect QTLs in diverse populations. Facilitated with high-throughput genotyping, agronomic QTLs and grain quality QTLs have been mapped by high-dense Single Nucleotide Polymorphism (SNP) markers through genome-wide association study (GWAS) ^22–25^. However, diverse population introduce population structure which could lead the spurious association if they are not accounted for^26,27^.

An alternative approach is to create multi-parental populations derived from elite parents in which each line represents a combination of alleles inherited from multiple parents. This allows the broadening of the genetic base and creates agronomically superior breeding lines through strategic recombination of genes/QTLs, thereby helps to select best lines suitable for targeted breeding programs. Multi-parent Advanced Generation Inter-crosses (MAGIC) populations have been developed in a number of crop species such as rice, corn, bread wheat, durum wheat, barley and chickpea^28^. A comprehensive review of the development and use of MAGIC populations has been provided^28^. Applications of MAGIC populations have been discussed and adopted within rice community to develop multi-parental populations^29–31^. MAGIC involves intercrossing a number of parental lines for “n” generations in a mating design to combine the genomes of all parents in the progeny lines. It can be used for coarse mapping with low marker densities on lines derived from an early generation and for fine mapping QTL using lines derived from more advanced generation^32^. In this study, QTL analysis on yield and related component traits, and grain quality traits was conducted in MAGIC global population (MGP) developed at the International Rice Research Institute (IRRI). The main objectives of the study were to identify the loci that were responsible for higher grain yield, superior agronomic characters, good grain quality and biofortification, and map the QTLs with higher resolution and study interactions. Based on the QTL identified, tightly linked SNP markers can be used by breeders for marker-aided selection to precisely introduce beneficial QTLs into elite lines for crop improvement.

## Results

### Trait variances and correlations

Nine traits (agronomical and biofortification traits) were measured in both 2015 Dry Season (2015DS) and 2016 Dry Season (2016DS), while 16 traits (agronomical, grain quality and biofortification traits) were measured in the 2016DS. MGP presented substantial variations for all traits during both 2015DS and 2016DS (Table S1). The results from 2015DS showed that among the parental lines CSR30 had the highest Best Linear Unbiased Estimator (BLUE) values for number of productive tillers (PTN), grain iron (Fe) and grain zinc (Zn). Inia Tacuari had the highest BLUE values for grain weight per panicle (GWT) and chlorophyll content index (SPAD) in flag leaf at maturity stage. Cypress, Samba Mahsuri + Sub1 and WAB 56–125 had highest BLUE values for grain yield (GYLD), grain number per panicle (TGN) and panicle length (PNL) respectively. Colombia XXI, IR45427-2B-2-2B-1-1, IR77186-122-2-2-3 and IR77298-14-1-2-10 were less than 110 cm. Four lines showed better GYLD than the highest parent (10.08 tons/ha) while 1010 lines were less than that parent. A total of 62 lines showed better GYLD than top check variety (7.12 tons/ha), whereas 952 lines were less than that variety. During 2016DS, among the parents Colombia XXI had the highest BLUE values for PNL, grain length (GL) and GWT, and Shan-Huang Zhan-2 had highest BLUE values for PTN and Fe content. IR73571-3B-11-3-K2 had highest BLUE values for GYLD and amylose content (AC) while IR4630-22-2-5-1-3 and IR45427-2B-2-2B-1-1 had highest BLUE values for grain width (GW) and CSR30 had the highest BLUE values for Zn content. A total of 60 lines showed higher GYLD than the top parent (8.40 tons/ha), whereas 1278 lines had lower than top parent. A total of 243 lines showed better GYLD than top check variety (6.44 tons/ha), whereas 1095 lines were less than top check variety.

Most of the parents flowered and matured early except Samba Mahsuri + Sub1. In the MAGIC RILs, the ranges and means for majority of traits were similar in both 2015DS and 2016DS trials. However, both means and ranges were higher for plant height (PHT), TGN, GWT, Zn and Fe during 2015DS, while PTN, SPAD and GYLD ranges were higher during 2016DS. But PNL range was higher in 2015DS and mean was higher in 2016DS. The genotypic variance for all the traits during both the seasons was highly significant (*p* < 0.0001). The quantile-quantile (QQ) analyses showed almost normal distributions for most of the measured traits. Combined BLUE analysis (Two-stage analysis in PBTools) was also significant for genotypic variance of nine common traits between two dry seasons. Combined BLUE values of nine common traits (2015DS and 2016DS) and BLUE values of seven traits (2016DS) were used to perform for further analyses. Several significant correlations were identified among different traits. Of 36 possible correlations, there were 21 positive and 15 negative correlations in 2015DS, whereas GWT was significantly correlated with PNL and TGN at *p* < 0.05. In 2016DS, 120 possible correlations, there were 54 negative and 66 positive correlations, whereas 18 (15 positive and 3 negative) were significant at  *p* < 0.05. At a level of significance (*p* < 0.05), GYLD were positively correlated with PHT, PNL, number of filled grains (FG) and GWT, and negatively with Zn (Fig. S1A,B).

### Population structure analysis and linkage disequilibrium (LD)

For this population, the log likelihood revealed by STRUCTURE gradually increased from *k = *1 to *k = *5 but no obvious optimum was observed. In contrast, the maximum of D*k* was observed at *k = *2, indicating that population can be divided into two subgroups (Fig. S2A). However, STRUCTURE did not identify any significant population structure as D*k* value was very low in MGP. Four principal components (PCs) were used to measure the variations in the population. The first PC explained 4.7% variations while the rest three PCs explained less than 1.5% variations. PC analysis showed no major clustering in the population although Jinbubyeo and Inia Tacuari were observed in counting of wide variations from the population (Fig. S2B). The LD analysis showed that there is extensive variability in the magnitude of allele frequency correlations (r^2^) reflecting variations in LD across chromosomes through 66,309 SNP markers. Average LD decay between 200–400 Kb were observed among intra-chromosomal marker pairs across different physical distance groups in the population at r^2^ ~0.24, about half of its initial values (Table S2). Therefore, this MGP has no population structure with lower LD across the genome, representing a useful genetic resource for genetic studies and fine mapping major effect QTLs and genes in rice.

### Genome-wide association study

**Figure 1:**
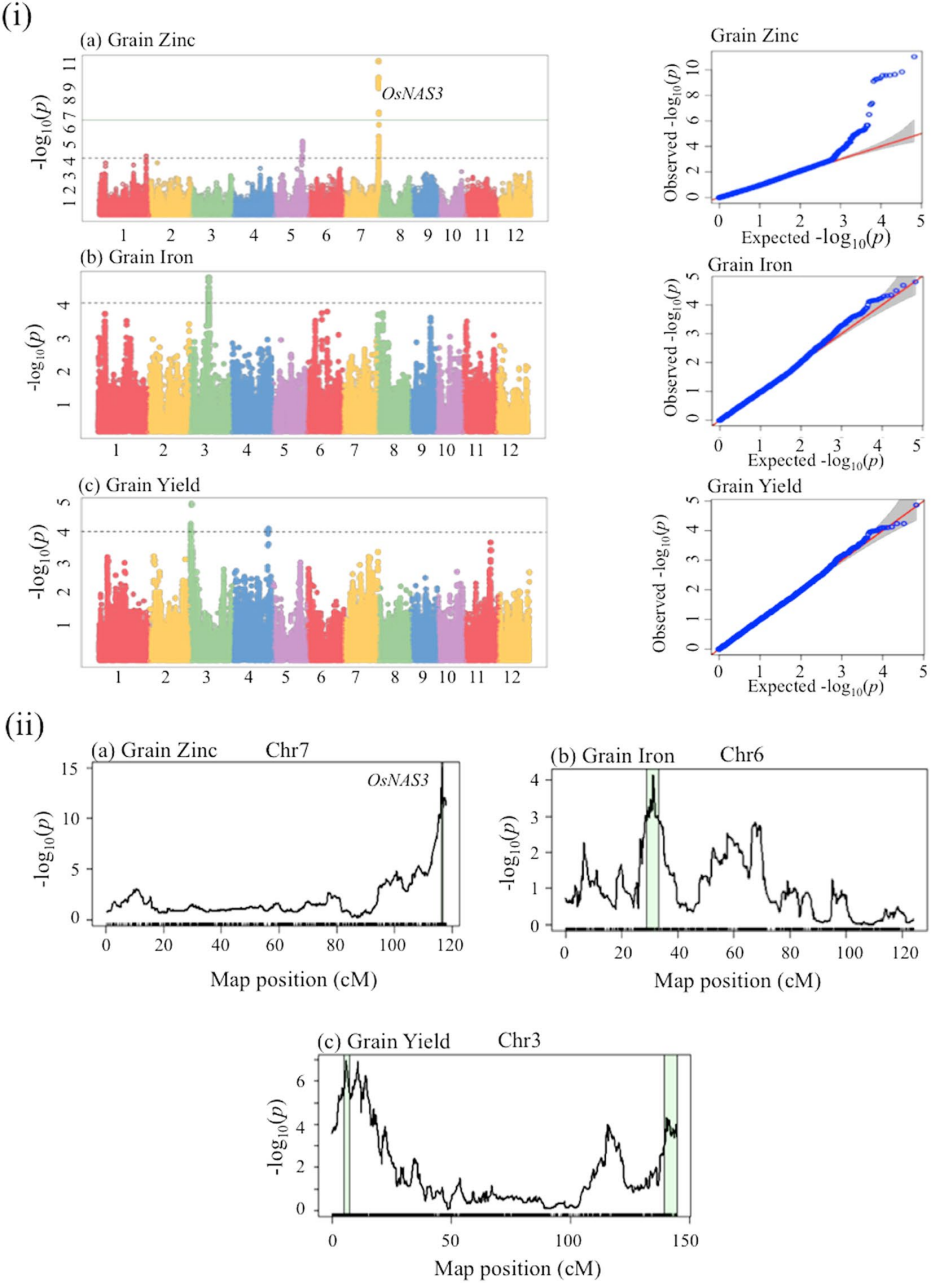
(i) Manhattan plots showing associated significant SNP markers for (**a**) grain zinc, (**b**) grain iron and (**c**) grain yield. X-axis shows chromosome number and Y-axis shows −log_10_(*p*). The horizontal line indicates threshold p-value at significant level (*p* < 0.0001). (ii) multi-parent interval mapping showing significant QTLs for (a) grain zinc on chromosome 7, (**b**) grain iron on chromosome 6 and (**c**) grain yield on chromosome 3. Light green colour indicates confident intervals of QTL regions.

Genome-wide association analysis (GWAS) was carried out to detect significant QTLs for 16 measured traits in MGP. A total of 1,027 MAGIC RILs, 16 parents and 66,309 SNP markers were used in association analysis. SNP makers significantly associated with different traits were detected at a threshold of  *p* < 0.0001. All the significant SNPs linked to a trait on a chromosomal region was considered as significant QTL or genomic region. The significant QTLs for each trait are provided (Figs. 1(i) and S3). A total of 38 QTLs were significantly associated with different traits and these QTLs were distributed on all chromosomes. The number of QTLs identified for each trait varied from 1 to 5. The highest number of QTLs were identified for GW and PNL on chromosome 1, 2, 3, 5, 7 and 8. For the remaining traits a maximum of three QTLs were identified. The phenotypic variance explained (PVE) by these QTLs varied from ~3.2 to 39.8% and 21 QTLs had PVE of more than 10%. In several QTL regions multiple SNPs were identified for different traits with clear peaks within wider confidence intervals while chalkiness (CHALKY), PTN and number of unfilled grains (UF) had one to two SNPs. Manhattan plots showed 25 significant QTLs for agronomic traits and 13 significant QTLs for grain quality and biofortication traits. The *qUF3* and *qCLK4* explained smallest QTL effects (PVE < 5%) for UF and CHALKY while *qPHT1* explained large QTL effect (PVE~40%) for PHT. Of 38 QTLs, 22 QTLs explained moderate to large QTL effect (PVE > 10%) for PHT, days-to-flowering (DTF), PNL, GL, GW, TGN, AC and Zn. The rest 16 QTLs explained small QTL effects (PVE < 10%) for PTN, SPAD, FG, UF, GWT, TGN, CHALKY and GYLD. In this study, GWAS identified a number of QTLs located either within or near reported genomic regions as well as newly detected QTLs across the genome. The QTL of plant height (*qPHT1*) was co-located with *qPH*_*1*_/*OsGA20ox2* underlying semi-dwarf trait while *qDTF3* and *qGYLD3* were located in very close proximity with major flowering activator genes (*qDF*_*3*_/*OsMADS50*, *Hd9*, *Hd1*) for DTF and GYLD traits. For grain quality QTLs *QGL3*, *qGW5* and *qAC6* were closely located with *GS3*, *qGW-5b* and *Wx* genes. Meanwhile, *QZn7* was co-located with *qZn*_*7*.*1*_/*OsNAS3*, long distance metal transporter for Zn (Table S3).

### Multi-parent interval mapping

Inia Tacuari and IR07F287 showed highest contributions of genomes among the parents. Cypress and Fedearroz50 were lowest in contributions of their genomes to the progenies (Fig. S4A). In genetic map, number of SNP markers varied from 342 on chromosome 9 to 845 on chromosome 1 (Fig. S4B). A total of 89 QTLs were identified for 16 traits from interval mapping (IM) at *p* < 0.0001 whereas the number of significant QTLs were reduced to 34 QTLs after fitting the full model (Figs. 1(ii) and S5). IM detected 19 QTLs for agronomic traits and 15 QTLs for grain quality and biofortification traits. Four QTLs *qPHT1*, *qDTF3*, *QGL3* and *qAC6* explained moderate to large QTL effects (PVE > 10%) for PHT, DTF, GL and AC. For PHT, *qPHT1* was detected on chromosome 1 with large QTL effect at PVE of 38.7%. PVE of three QTLs *qDTF3*, *QGL3* and *qAC6* explained moderate QTL effects and varied from 14.11–22.43% for DTF, GL and AC. The remaining 30 QTLs explained small QTL effects and varied from 2.37 to 8.72%. Two QTLs *qGYLD2* and *qGYLD3* varied from 2.8 to 4.41% for GYLD while *QZn1*.*1* and *QZn7* were from 5.33 to 7.71% for Zn. The *qUF2* QTL explained the smallest QTL effect (PVE~3%) for UF. Notably, major QTLs detected in IM were consistent with the QTLs uncovered by GWAS. These major reported QTLs *qPH*_*1*_*/OsGA20ox2*, *qDF*_*3*_*/OsMADS50*, *Hd9*, *Hd1*, *GS3*, *qGW-5b*, *Wx* and *qZn*_*7*.*1*_*/OsNAS3* were closely identified by IM for PHT, DTF, GYLD, GL, GW, AC and Zn traits (Table S4).

### Bayesian genomic prediction network

**Figure 2:**
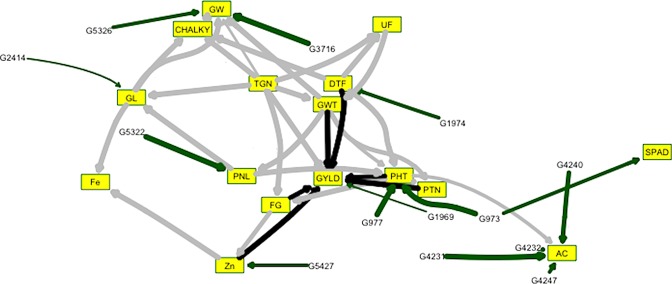
Bayesian networking showing trait-trait and trait-marker relationships for 16 traits. Yellow boxes represent the traits whereas black arrows show relationships between yield and other related traits. Green arrows show relationships between traits and markers which co-located with reported QTLs whereas strength of relationships is indicated by thickness of arrows. PHT, plant height; PTN, number of productive tillers; DTF, days-to-flowering; PNL, panicle length; SPAD, chlorophyll content index; FG, number of filled grains; UF, number of unfilled grains; GWT, grain weight per panicle; TGN, grain number per panicle; GYLD, grain yield; GW, grain width; GL, grain length; CHALKY, chalkiness; AC, amylose content; Zn, grain zinc and Fe, grain iron.

Bayesian Genomic Prediction Network (BN) explained that causal predictive correlations showed higher predictive power than genetic predictive correlations for all traits (Table 1). Moreover, BN showed the strength and direction of relationships among traits and markers (Fig. S6). A total of 60 QTLs were identified by BN whereas 31 QTLs were agronomic traits and 29 QTLs for grain quality and biofortification. BN consistently identified major reported QTLs, uncovered by GWAS and IM *qPH*_*1*_*/OsGA20ox2*, *qDF*_*3*_*/OsMADS50*, *GS3*, *qGW-5b*, *Wx* and *qZn*_*7*.*1*_*/OsNAS3* for PHT, DTF, GYLD, GL, GW, AC and Zn traits (Fig. 2; Table S5). Further, a total of 73 nodes and 119 associations were observed in BN of 16 traits. There were 22 marker-marker associations, 32 trait-trait associations and 65 marker-trait associations in BN analysis. At averaged BN (Strength > 0.5), significant direct associations among the traits were PHT~PNL:GWT:DTF, PTN~PHT:GWT, PNL~GWT, UF~TGN:DTF, GWT~TGN:UF, FG~PHT:TGN, GYLD~Zn:PHT:PTN:TGN: GWT:FG:DTF, GW~TGN:GWT:GL, GL~PNL:TGN, AC~PHT, CHALKY~TGN:DTF:GL:GW, Zn~FG and Fe~Zn:GL. At significant marker-trait associations, numbers of significant markers varied from one to eight markers for respective traits. GW and Zn were associated with eight markers for each trait while only one marker associated with FG.

**Table 1:** Genetic and causal predictive correlations for 16 traits using BN analysis at α = 0.01. (GPC: Genetic Predictive Correlation, CPC: Causal Predictive Correlation).

	GYLD	Zn	Fe	PHT	PTN	PNL	TGN	GWT	SPAD	FG	UF	DTF	GL	GW	CHALKY	AC
GPC	0.20	0.37	0.15	0.61	0.15	0.23	0.11	0.10	0.13	0.08	0.07	0.23	0.37	0.41	0.06	0.46
CPC	0.37	0.39	0.25	0.69	0.24	0.47	0.23	0.73	0.13	0.38	0.19	0.24	0.46	0.47	0.27	0.46

### Candidate QTLs/Genes analysis

**Figure 3:**
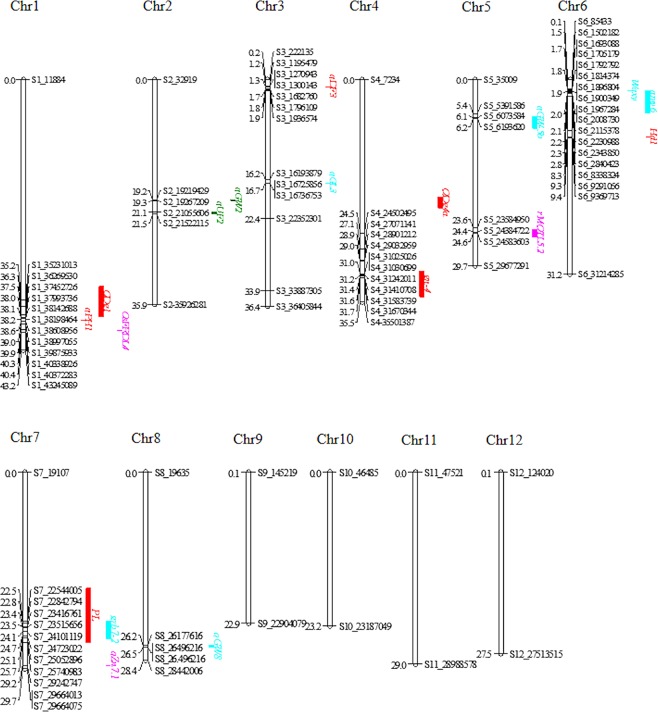
Locations of the consistent QTLs across the chromosomes identified by either two or three analyses (GWAS, IM and BN) in MAGIC global population. All the QTLs for agronomic traits highlighted in red, for grain quality in light blue (turquoise), for biofortification in bubble and novel QTLs are in green.

Candidate genes analysis was carried out using peak SNP markers detected in at least two of three analyses (GWAS, IM and BN). All known genes and fine mapped QTLs of the significant markers were shortlisted in Table 2. Ten QTLs *qPHT1*, *qDTF3*, *qPNL7*, *qCHP1*, *qGW5*, *qGW7*, *QGL3*, *qAC6*, *QZn7* and *qGYLD3* were consistently identified in three analyses whereas *qDTF6*, *qCHP4*, *qUF2*, *qGN4*, *qGW2*, *qGW3*, *qGW8*, *QZn1* and *QZn5* were identified in at least two analyses. For reported QTLs of these genomic regions, plant height QTL *qPHT1* was in close proximity with *qPH*_*1*_*/OsGA20ox2*, semidwarf gene on chromosome 1. Two flowering QTLs *qDTF3* and *qDTF6* were in close proximity with *qDF*_*3*_*/OsMADS50*, flowering activator gene and *Hd1* on chromosome 3 and 6. With co-location of QTLs, flowering QTL *qDTF3* and grain yield QTL *qGYLD3* were co-located with *qDF*_*3*_*/OsMADS50* gene on chromosome 3. Panicle length QTL *qPNL7* was positioned within *PL* on chromosome 7 while *qCHP1* and *qCHP4* were co-located with *QDg1* and *QDg4a* on chromosome 1 and 4 for chlorophyll content index. The grain number QTL *qGN4* was co-located with *gn-4* on chromosome 4 while *qUF2* was novel QTL for unfilled grain on chromosome 2. Grain width QTLs *qGW3*, *qGW5* and *qGW7* were co-located with *qGL*_*3*_*/GS3*, *qGW-5b*, and *grb7-2* on chromosome 3, 5 and 7 whereas *qGW2* and *qGW8* have not reported in QTLs databases. Grain length *QGL3* was positioned within the *qGL*_*3*_*/GS3* gene, underlying grain shape on chromosome 3. For grain quality and biofortification, *qAC6* was positioned within *Amy6/Wx* gene on chromosome 6 for AC whereas *QZn1*, *QZn5* and *QZn7* were co-located with metal transporter genes *OsFRDL4*, *rMQTL5*.*2* and *OsNAS3* on chromosome 1, 5 and 7 for Zn (Fig. 3). In gene association analysis, ten candidate genes were identified for GYLD on chromosome 3 whereas 78 candidate genes were identified on chromosome 6 for AC. A total of 22 candidate genes were associated with Zn on chromosome 7 while 10 candidate genes were associated with Zn on chromosome 5. All the top five candidate genes of grain yield, grain quality and biofortification traits were shortlisted in Table S6.

**Table 2:** Consistent QTLs/Genes detected in at least two of the three analyses (GWAS, IM mapping and BN).

Trait	Detected QTLs (Term)	SNP	Chr	PVE (%)	Reported QTLs	Start	End	DB
PHT	*qPHT1*	S1_38286772	1	39.85	*qPH*_*1*_ *(OsGA20ox2)*	38382385	38385469	RAP DB
DTF	*qDTF3*	S3_1270943	3	28.28	*qDF*_*3*_ *(OsMADS50)*	1270320	1300273	RAP DB
	*qDTF6*	S6_8338324	6	10.43	*Hd1*	9282505	9327178	Gramene
PNL	*qPNL7*	S7_24669663	7	12.28	*PL*	17525817	25775868	Gramene
SPAD	*qCHP1*	S1_38244911	1	12.34	*QDg1*	32987234	37889506	Gramene
	*qCHP4*	S4_19858550	4	3.75	*QDg4a*	18824746	20519179	Gramene
UF	*qUF2*	S2_21055606	2	2.90	—	—	—	—
TGN	*qGN4*	S4_31250082	4	6.78	*gn-4*	30630093	34698383	Gramene
GW	*qGW2*	S2_19219429	2	16.25	—	—	—	—
	*qGW3*	S3_16738452	3	16.88	*qGL*_*3*_	16729501	16735109	RAP DB
	*qGW5*	S5_5391586	5	17.17	*qGW-5b*	5915709	7810160	Gramene
	*qGW7*	S7_24575488	7	17.77	*grb7-2*	22532352	25188107	qtaro
	*qGW8*	S8_26496216	8	15.91	—	—	—	—
GL	*QGL3*	S3_16790082	3	16.67	*qGL*_*3*_*/GS3*	16729501	16735109	RAP DB
AC	*qAC6*	S6_1760469	6	19.25	*amy6/Wx*	1764586	5425631	Gramene
Zn	*QZn1*	S1_40372091	1	17.57	*OsFRDL4*	40,093,456	40,097,016	RAP DB
	*QZn5*	S5_24312726	5	17.95	*rMQTL5*.*2*	23906571	25164524	Jin, T. *et al*. (2015)
	*QZn7*	S7_29281096	7	20.10	*qZn*_*7*.*1*_ *(OsNAS3)*	29323098	29324607	RAP DB
GYLD	*qGYLD3*	S3_1222496	3	9.21	*qDF*_*3*_ *(OsMADS50)*	1270320	1300273	RAP DB

(*qPHT1*, *qDTF3*, *qPNL7*, *qCHP1*, *qGW5*, *qGW7*, *QGL3*, *qAC6*, *QZn7* and *qGYLD3* QTLs were detected by all approaches).

### MAGIC lines with multiple QTLs pyramided

In MAGIC global population, phenotypic analysis showed wider variations for 16 traits during dry seasons. QTL combinations were observed in MAGIC RILs from the contributions of 16 founders. Reshuffling of these founder genomes increased crossovers to break negative drag effects between two genetic loci. Out of 1,027 RILs, 72 lines were found with high GYLD and Zn. Meanwhile, 69 lines were observed with high GYLD and Zn, and early flowering (DTF), 18 lines with high GYLD and Zn (~18 ppm), early flowering (DTF), taller plants (PHT) and moderate AC. Correspondent QTLs and allelic combinations are being further investigated for these pyramided RILs. Based on acceptable yield and zinc level, ten best multi-trait pyramided RILs have been shortlisted and presented in Table 3. These promising lines with multiple trait combinations will provide a good genetic resource for breeding programs.

**Table 3:** MAGIC RILs that posses combinations of high yield, agronomic traits, grain quality and biofortification.

Line ID	GYLD (tons/ha)	Zn (ppm)	Fe (ppm)	DTF (days)	PHT (cm)	FG (no.)	GWT (grams)	GL (mm)	GW (mm)	CHALKY (%)	AC (%)
MG.8312	7.51	19.10	4.76	87.21	133.28	515.53	2.82	6.49	2.38	7.60	25.66
MG.8161	7.23	18.12	5.56	83.69	140.96	191.56	1.64	6.01	2.24	2.33	24.34
MG.8264	7.22	18.41	4.28	83.81	133.34	228.89	3.82	6.42	2.04	0.90	24.76
MG.7604	7.21	18.17	4.36	91.15	133.00	325.44	2.89	5.68	2.20	3.21	26.24
MG.7102	7.13	18.64	3.92	93.15	134.96	266.44	2.02	NA	NA	NA	20.34
MG.8358	6.68	20.52	3.73	87.21	119.24	348.53	2.71	6.23	2.08	8.75	21.06
MG.7418	6.62	18.52	3.70	NA	134.31	252.44	2.71	6.45	2.17	3.61	24.64
MG.7117	6.61	19.56	2.73	91.15	132.37	289.04	3.08	5.85	2.12	1.04	22.79
MG.7921	6.53	20.06	4.52	84.15	135.52	434.04	3.40	6.61	1.92	3.60	24.69
MG.8354	6.53	18.19	6.02	87.21	112.61	368.53	3.41	5.40	2.12	3.52	23.76

## Discussion

Most of the economically important traits in rice are quantitatively inherited in genetic manner^33^. Combination of association and pedigree-based studies was a good approach to identify small and large effect QTLs using appropriate mapping population. In previous studies, most of mapping populations have been limited to apply both association and pedigree-based studies^14,15,21,22,26,34,35^. MAGIC global population is a unique genetic resource with wider genetic diversity representing *indica* and *japonica* subgroups without prominent population structure as well as low LD^28–31,36^. Phenotypic analysis showed substantial variations for 16 measured traits and transgressive RILs for further genetic analysis. In a Pearson correlation, we observed positive correlations between GYLD and, PHT, PNL, FG and GWT, and negatively with Zn. Meng’s group reported that population structure in MAGIC population was negligible as an intercrossed population^37^. Our study suggests that no major clustering was observed by STRUCTURE and PC analyses. The LD decay distance is an important factor in determining the association mapping resolution as high LD decay enhance the fine mapping of QTL regions^38^. Different LD decay rates of MAGIC rice populations have been reported by previous studies^37,39,40^. The results of LD decay showed high rate of recombination with an average LD decay around 300 kb (r^2^ = 0.24). High LD decay increased mapping resolution whereas non-significant population structure reduced spurious marker-trait association^28,30,31,40^.

In this study, we used a unique mapping population with large population size, adequate marker density and appropriate statistical model to detect significant QTL regions though different SNP marker sets used for different analyses based on statistical model and computational power. Significant marker-trait associations and interactions were captured through the association and pedigree-based analyses. All analyses (GWAS, IM, BN) have identified significant QTLs in close proximity with known QTLs/genes *qPH*_*1*_/*OsGA20ox2*, *qDF*_*3*_/*OsMADS50*, *PL*, *QDg1*, *qGW-5b*, *grb7-2*, *qGL*_*3*_/*GS3*, *Amy6*/*Wx* gene and *qZn*_*7*.*1*_/*OsNAS3* for PHT, DTF, GYLD, PNL, SPAD, GW, GL, AC and Zn across the genome (https://rapdb.dna.affrc.go.jp/; https://archive.gramene.org/qtl/; http://qtaro.abr.affrc.go.jp/). These results indicate that all analyses used the validity and appropriateness of model for the study. Aside from these QTLs, we also detected unknown and known QTLs across the genomes in either one or two analyses. Based on differences in statistical performance, each analysis can detect the QTL that was not detected by other analyses. However, these QTLs still require further validations before they can be incorporated in breeding program.

Our study is a first report for exploring genetic architecture of grain yield and grain quality through the combination of association and pedigree-based studies in 16-way MAGIC rice population although several studies reported for yield and grain quality traits^1,3,4,8,9,11–13^. Many published studies mentioned that most of high-yielding varieties have longer growth duration for longer metabolic activities and grain filling^41^. In this study, GYLD and DTF were co-located with *qDF*_*3*_/*OsMADS50*, flowering activator genes on chromosome 3. This result suggests that there is a pleiotropic interaction between GYLD and DTF, consistent with previous studies^31,41^. Further, we explored the interactions among yield and quality traits through the BN prediction. BN prediction revealed that PHT, DTF, GWT, TGN, PTN, Zn and FG were directly associated with GYLD. Consistent with previous reports, we detected negative correlations between GYLD and, DTF and Zn^40–42^. Low recombination rate in bi-parental population is a limiting factor to break the negative drag effects among the traits^21^. However, reshuffling of 16 founder genomes help breaking the negative drag effects between two genetic loci in the population. For instance, we are able to select pyramided lines which have high yield with short lifespan, and high yield with high zinc content.

In conclusions, MAGIC global population provided a valuable genetic resource with multi-trait combinations. The promising lines with multiple traits will make them ideal for direct utilization in breeding. With a unique population, combination of association and pedigree-based studies was a powerful tool to identify significant candidate QTLs as well as interactions among the traits. In this study, we uncovered candidate QTLs with high mapping resolution, interval regions of candidate QTLs, marker-marker associations, marker-trait associations and the trait-trait associations of 16 measured traits. Consistent significant markers identified in all analyses can be directly used in MAS to facilitate screening the breeding lines with desirable traits in crop improvement programs. The validation of novel regions and candidate genes will be a focus of future research.

## Methods

### MAGIC global population

**Figure 4:**
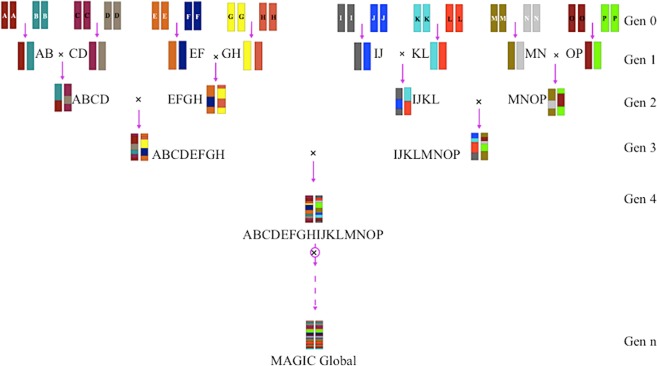
Development of MAGIC global population (MGP). MGP was produced by using 16 founders representing *indica* and *japonica* pools through multiple crosses and selfing. “A-P” letters represent 16 founders in MGP.

The MAGIC *indica* and *japonica* populations were developed at IRRI by using eight elite founders from *indica* pool and eight elite founders from *japonica* pool. These founders possessed good grain quality, high yield potential, biotic and abiotic stress tolerance. Both MAGIC populations followed the same scheme of development^29^. Here, MAGIC global population was developed by expanding the diversity to increase recombination between the eight *indica* and eight *japonica* MAGIC pools through additional cycles of intercrossing. The eight-way F_1_’s derived during the development of the MAGIC *indica* population were crossed to the eight-way F_1_’s derived during the development of the MAGIC *japonica* population. A total of 150 sixteen-way crosses were advanced for a number of selfing generations (S_8_) to create MAGIC global population. Therefore, MAGIC global population is representative of 16 founders of *indica* and *japonica* pools (Fig. 4).

### Field trials and trait measurements

MAGIC global population was grown during 2015DS and 2016DS at IRRI. We followed standard field management practices to raise good crop^43^. During ripening stage (about 30 days after flowering), 9 traits (agronomic and biofortification traits) and 16 traits (agronomic and grain quality traits including biofortification traits) were measured in 2015DS and 2016DS. In 2015DS trial, three uniform plants in the middle of each plot were measured for PHT, PTN, SPAD at maturity and three panicles harvested from each plot were sampled to measure PNL, GW and TGN. The inner twelve hills (3 × 4) were harvested for measurement of GYLD and adjusted 14% moisture content. In 2016DS, seven additional traits such as DTF, FG, UF, GW, GL, CHALKY and AC were measured. In 2016DS, about 30–40 hills were harvested for GYLD after removing last border row. Yield per plot was converted to tons/ha^31^. Zn and Fe were measured by using milled rice in both dry seasons. AC was measured by using Skalar San++ System Segmented Flow Analyser (SFA) which consists of an autosampler, an amylose chemistry unit (manifold, proportioning pump and colorimeter with 620 nm filter). Grain physical appearances (GW, GL and CHALKY) were measured by using SeedCount SC5000 Image Analyzer. For measuring grain Zn and Fe, milled rice samples weighting at least 3 g were subjected to X-ray fluorescence (XRF) analysis using Bruker S_2_ Ranger for Zn and Fe. Measurements were done twice per sample and was expressed in parts per million (ppm).

The statistical analyses of all measured traits were performed using PB Tools software (http://bbi.irri.org/) and R/Asreml. For nine common traits of both dry seasons, adjusted means from P-rep and AugRCB designs were first weighted by 1/mse. The weighted means were used to perform combined analysis in a two-stage analysis within PB Tools software based on error mean square (mse), standard error and number of replicates. Statistical significance of seven additional measured traits from 2016DS were analysed by using AugRCB design in R/Asreml. Correlations, boxplot and basic statistical parameters were calculated in R programs. Skewed phenotypic data was normalized by using rankTransPheno function in R/FRGEpistasis program. A total of 1027 common genotypes between two dry seasons and parents were used to perform GWAS, IM and BN.

### Genotypic assay

#### Genotyping by sequencing (GBS) and SNP calling

About 2 milligrams leaf samples of 1330 genotypes with replicates were collected by using PlantTrak Hx sampling method. DNA extraction was conducted by using oKtopure Extraction protocol in the Genotyping Service Laboratory at IRRI. DNA library was sent to Cornell University for SNP multiplex analysis using Illumina’s GBS protocol^44^. The GBS pipeline was run by the Philippine Genome Center of the University of the Philippines using Tassel software Version 3.0.169^45^. The sequence reads were aligned to the reference genome Nipponbare sequence MSUv7 to derive the physical positions of markers. Post-processing steps were applied to the genotype data for generating quality SNPs by imposing various criteria^31^. After filtering post-GBS pipeline, different SNP datasets were generated for multiple approaches. A 22,338 SNP markers  were generated for pedigree-based analysis after filtering parents at minor allele frequency (MAF) (1/16) with no missing data while 66,309 SNP markers were generated for association analysis at MAF (0.05) and call rate (70%). From the 22,338 SNP markers, 8,110 SNP markers were extracted for BN analysis based on MAF (0.05), r^2^ < 0.5 and no heterozygous call while 6,170 SNP markers were binned and extracted for genetic mapping at no closer than 0.1 cM (Fig. S7).

#### Population structure analysis and linkage disequilibrium

Population structure was performed by 8110 SNP markers using a model based Bayesian clustering analysis method, implemented in STRUCTURE software Version 2.3.4^46^. The program was run with the following parameters: k, the number of groups in the panel varying from 1 to 5; 10 runs for each k value; for each run, 10,000 burn in iterations followed by 10,000 MCMC (Markov Chain Monte Carlo) iterations. The optimal number of K clusters was estimated with the parameter (ΔK) of^47^ in Structure Harvester^48^. In addition, four PCs were conducted for population analysis by using 66,309 SNP markers through R/SNPRelated package. The results of clustering in the population were interpreted based on percent variations explained by different PCs. The intra-chromosomal linkage disequilibrium (LD) between SNP marker pairs were calculated by r^2^ values between the pairs of markers using 66,309 SNPs in TASSEL v5.2.20. Marker pairs with statistically significant LD (pDiseq < 0.05) were considered in the LD decay analysis. The LD decay rate was measured as the average r^2^ dropped to half of its maximum value^12,24^.

#### Genome-wide association study

A genome-wide association study (GWAS) was performed for 16 traits using 66,309 SNPs and mean BLUEs of each trait. All statistical analyses were performed using the PBTools and R/Asreml software packages (Fitting linear mixed model using residual maximum likelihood, Version 3.0). GWAS was carried out using R/GAPIT (Genome Association and Prediction Integrated Tool)^49^. The compressed mixed linear model (MLM) method was applied for detecting QTL associated with the trait. This MLM allowed correction to cryptic relatedness and other fixed effects using a kinship matrix and population stratification through principle components^50^. The default criteria implemented in GAPIT was used with a significance threshold of *p* < 0.0001.

#### Multi-parent interval mapping

Multi-parent interval mapping was carried out for 16 traits using 6,170 SNP markers. Founder probabilities of 16 parents and percentage of recombination per chromosome were estimated using R/Happy Version 2.3. The genetic map of the population was generated by using 6,170 SNP markers at average marker density at ~63 Kb through R/mpMap. Significant QTLs were detected by conducting interval mapping using the functions ‘mpprob’ and ‘mpIM’ through R/happy and R/mpMap^51^. Simple interval mapping (SIM) was carried out using adjusted means as response. A QTL was considered as important in SIM after passing a significance threshold level at *p* < 0.0001. The effects of all QTLs were used to simultaneously estimate from the function ‘fit’ by fitting all the detected QTLs in a single model or full model (both fixed and random effects).

#### Bayesian genomic networking

The averaged Bayesian network in multiple QTLs analysis was conducted by using 8,110 SNP markers for 16 traits following the instructions of Scutari’s group^52^. The package lme4 was used to adjust for family structure while bnlearn was used to learn the model and perform predictions, and parallel to speed up learning. We encoded short labels to the marker names after preprocessing data file. Moreover, we identified which variables in the data are traits, which are markers, which contain variety IDs and pedigree information. The Bayesian network model was fitted by the ‘fit.the.model()’ function which takes the data and the type I error threshold alpha to use for structure learning as arguments. The type I error alpha was set at 0.01 in this study.

#### Candidate QTLs/genes analysis

Candidate QTLs/genes were identified using publicly available databases; RAP DB (https://rapdb.dna.affrc.go.jp/), QTARO (http://qtaro.abr.affrc.go.jp/) and GRAMENE (https://archive.gramene.org/qtl/) databases. All candidate QTLs/genes of significant genomic regions were searched to provide additional insight in genetic architecture of grain yield and grain quality traits using annotated Napponbare reference genome (MSUv7) through Galaxy/IRRI Bioinformatics (http://galaxy.irri.org/). Within ±200 kb (100 kb - SNP + 100 kb) of the peak SNP, gene association analysis was carried out for GYLD, AC and Zn using MAGMA Version 1.06 for detecting significant candidate genes.

## Supplementary information

Supplementary Information

Supplementary Information
