# Supplementary material for: Exploring genetic architecture of grain yield and quality traits in a 16-way indica by japonica rice MAGIC global population
Source: Sci Rep. 2019 Dec 20;9:19605. doi: 10.1038/s41598-019-55357-7 (PMC6925145; doi:10.1038/s41598-019-55357-7)
Supplement: Supplementary file 1 — Supplementary Information [file 41598_2019_55357_MOESM1_ESM.pdf]

Exploring genetic architecture of grain yield and quality traits in a 16-way *indica* by *japonica* rice MAGIC global population

Hein Zaw[1,3], Chitra Raghavan[1], Arnel Pocsedio[1], B.P. Mallikarjuna Swamy[1], Mona Liza Jubay[1], Rakesh Kumar Singh[4], Justine Bonifacio[1], Ramil Mauleon[1], Jose E. Hernandez[2], Merlyn S. Mendioro[2], Glenn B Gregorio[1,2], Hei Leung*[1]

Supplementary Table S1: Phenotypic variations in 16 traits of MAGIC global population in two dry seasons.

Trait	Range	Mean	Stdev	Dry seasons
PHT(cm)	77.47 – 185.12	113.37	3.58	2015DS
PHT(cm)	71.60 – 169.10	110.35	4.19	2016DS
PTN(no.)	6 – 37.67	11.3	1.87	2015DS
PTN(no.)	7 – 43.3	17.3	2.42	2016DS
PNL(cm)	18 – 36.33	23.64	0.77	2015DS
PNL(cm)	13.57 – 34.7	24.55	1.76	2016DS
SPAD	1.5 – 19.4	4.37	1.43	2015DS
SPAD	0.72 – 25.80	5.25	2.1	2016DS
DTF(day)	70.69 – 115.80	88.33	1.92	2016DS
GYLD(tons/ha)	1.26 – 12.65	4.79	0.73	2015DS
GYLD(tons/ha)	1.28 – 14.52	4.97	1.01	2016DS
FG(no.)	22.81 – 222.29	94.53	25.57	2016DS
UF(no.)	2.6 – 135	25.89	17.81	2016DS
TGN(no.)	24.46 – 296.79	150.65	12.36	2015DS
TGN(no.)	29.15 – 285.30	96.65	33.37	2016DS
GWT(gram)	1.03 – 5.83	2.96	0.23	2015DS
GWT(gram)	0.54 – 5.70	1.91	0.48	2016DS
AC(%)	11.96 – 28.64	21.8	0.84	2016DS
GL(mm)	4.69 – 7.67	5.79	0.18	2016DS
GW(mm)	1.74 – 2.73	2.16	0.08	2016DS
CHALKY(%)	0.016 – 50.84	2.97	3.58	2016DS
Zn(ppm)	9.7- 31.27	17.17	1.96	2015DS
Zn(ppm)	9.22 – 26.83	14.52	1.08	2016DS
Fe(ppm)	0.99 – 11.62	4.72	0.86	2015DS
Fe(ppm)	0.4 - 11	3.47	0.91	2016DS

Supplementary Table S2: Linkage disequilibrium statistics in MAGIC global population using 66,309 SNP markers.

**Distance**	**Mean r^2 of significant LD marker pairs**	**Number of significant LD marker pairs**	**% significant LD in r^2>0.2**	**Mean r^2 in Marker Pairs (r^2>0.2)**	**Marker Pairs in complete LD (r^2>0.9)**
0 – 5kb	0.4976564	163105	101607	0.7631035	53787
5 – 50kb	0.3813683	654404	347782	0.6661668	108191
50 – 100kb	0.3269357	667912	321507	0.6159242	59468
100 - 200kb	0.283672	1291241	563045	0.5761866	58916
200 - 400kb	0.237557	2523227	971233	0.5273503	43738
400 - 600kb	0.1974864	2465354	816898	0.4857807	18824
600 - 800kb	0.1710361	2415144	707345	0.4568373	12280
800 - 1000kb	0.1532413	2368339	629529	0.4348107	9002
1 - 1.2Mb	0.1376961	2321059	553478	0.4208944	7698
1.2 - 1.4Mb	0.1238276	2286020	481675	0.4089487	6243
1.4 - 1.6Mb	0.1133103	2265766	433459	0.3966835	5824

Supplementary Table S3. Candidate QTLs and co-located QTLs detected by GWAS for 16 traits using MAGIC global population.

Trait	SNP	Chr	Pos	P.value	PVE (%)	Detected QTLs (Term)	Reported QTLs	Start	End	DB
**PHT**	S1_38286772	1	38286772	3.18E-38	39.8	*qPHT1*	qPH_1	38,382,385	38,385,469	RAP DB
	S1_38292649	1	38292649	1.26E-31	37.4	*qPHT1*	qPH_1	38,382,385	38,385,469	RAP DB
	S1_38433096	1	38433096	6.31E-31	37.1	*qPHT1*	qPH_1	38,382,385	38,385,469	RAP DB
	S3_1195479	3	1195479	2.19E-05	28.4	*qPHT3*	*Hd9*	975995	1427051	qtaro
	S3_1682760	3	1682760	4.69E-05	28.3	*qPHT3*	*Hd9*	975995	1427051	qtaro
	S3_1270943	3	1270943	5.12E-05	28.3	*qPHT3*	*Hd9*	1,270,320	1,300,273	RAP DB
**PTN**	S4_31162467	4	31162467	5.68E-05	8.9	*qTn4*	*Tn*	24,732,111	31,065,616	Gramene
	S4_31215525	4	31215525	9.51E-05	8.8	*qTn4*	*Tn*	24,732,111	31,065,616	Gramene
**DTF**	S3_1270943	3	1270943	1.71E-17	15.5	*qDTF3*	qDF_3	1,270,320	1,300,273	RAP DB
	S3_1195479	3	1195479	1.03E-16	15.2	*qDTF3*	*Hd9*	975995	1427051	qtaro
	S3_1796079	3	1796079	1.72E-13	13.8	*qDTF3*	*Hd9*	975995	1427051	qtaro
	S6_8338324	6	8338324	1.80E-05	10.4	*qDTF6*	*Hd1*	9,282,505	9,327,178	Gramene
	S6_8338327	6	8338327	1.80E-05	10.4	*qDTF6*	*Hd1*	9,282,505	9,327,178	Gramene
	S6_8338333	6	8338333	1.80E-05	10.4	*qDTF6*	*Hd1*	9,282,505	9,327,178	Gramene
**PNL**	S1_34704181	1	34704181	1.27E-05	12.3	*qPNL1*	*qPL-1*	34,032,918	34,940,769	Gramene
	S1_38440552	1	38440552	7.91E-06	12.4	*qPNL1.1*	qPH_1	38,382,385	38,385,469	RAP DB
	S1_38244911	1	38244911	1.04E-05	12.3	*qPNL1.1*	qPH_1	38,382,385	38,385,469	RAP DB
	S3_8548868	3	8548868	9.64E-05	12.0	*qPNL3*	*gl3a*	8460597	9806441	qtaro
	S7_24669663	7	24669663	1.53E-05	12.3	*qPNL7*	*PL*	17,525,817	25,775,868	Gramene
	S7_24669596	7	24669596	1.70E-05	12.3	*qPNL7*	*PL*	17,525,817	25,775,868	Gramene
	S7_24645555	7	24645555	2.58E-05	12.2	*qPNL7*	*PL*	17,525,817	25,775,868	Gramene
	S7_28960591	7	28960591	6.17E-05	12.0	*qPNL7.1*	*fzp*	28959761	28961248	qtaro
**SPAD**	S1_38244911	1	38244911	3.57E-08	4.8	*qCHP1*	*QDg1*	32,987,234	37,889,506	Gramene
	S1_38207165	1	38207165	8.58E-08	4.7	*qCHP1*	*QDg1*	32,987,234	37,889,506	Gramene
	S1_38286772	1	38286772	2.07E-07	4.5	*qCHP1*	*QDg1*	32,987,234	37,889,506	Gramene
	S4_19858550	4	19858550	1.14E-05	3.8	*qCHP4*	*QDg4a*	18,824,746	20,519,179	Gramene
	S4_19895568	4	19895568	4.98E-05	3.5	*qCHP4*	*QDg4a*	18,824,746	20,519,179	Gramene
	S4_19850161	4	19850161	4.78E-07	4.3	*qCHP4*	*QDg4a*	18,824,746	20,519,179	Gramene
**FG**	S4_31133351	4	31133351	1.98E-06	6.0	*qFG4*	*gn-4*	30,630,093	34,698,383	Gramene
	S4_31127313	4	31127313	4.32E-06	5.8	*qFG4*	*gn-4*	30,630,093	34,698,383	Gramene
	S4_31178256	4	31178256	4.51E-06	5.8	*qFG4*	*gn-4*	30,630,093	34,698,383	Gramene
	S4_16160917	4	16160917	3.43E-05	5.5	*qFG4.1*	-	-	-	-
	S8_10181188	8	10181188	9.73E-05	5.3	*qFG8*	-	-	-	-
**UF**	S3_1270943	3	1270943	6.97E-05	3.7	*qUF3*	*QSs3*	1,449,758	3,236,745	Gramene
	S3_22283352	3	22283352	4.04E-05	3.8	*qUF3.1*	*qGW-3*	18350837	22306369	qtaro
**GWT**	S4_24502495	4	24502495	2.73E-06	6.5	*qGWT4*	*gpp4*	21645173	34244441	qtaro
	S4_25832541	4	25832541	5.92E-06	6.3	*qGWT4.1*	*gpp4*	21645173	34244441	qtaro
	S4_27071141	4	27071141	1.85E-06	6.5	*qGWT4.1*	*gpp4*	21645173	34244441	qtaro
	S4_31050939	4	31050939	2.43E-07	6.9	*qGWT4.2*	*gn-4*	30,630,093	34,698,383	Gramene
	S4_31025026	4	31025026	3.53E-07	6.8	*qGWT4.2*	*gn-4*	30,630,093	34,698,383	Gramene
	S4_31250082	4	31250082	4.53E-07	6.8	*qGWT4.2*	*gn-4*	30,630,093	34,698,383	Gramene
**TGN**	S1_42038899	1	42038899	1.41E-05	8.5	*qGN1*	*QGn1*	41,967,890	41,969,197	Gramene
	S4_31250082	4	31250082	1.22E-09	10.2	*qGN4*	*gn-4*	30,630,093	34,698,383	Gramene
	S4_31050939	4	31050939	1.62E-09	10.1	*qGN4*	*gn-4*	30,630,093	34,698,383	Gramene
	S4_31243064	4	31243064	2.53E-09	10.1	*qGN4*	*gn-4*	30,630,093	34,698,383	Gramene
**GW**	S2_19219429	2	19219429	7.90E-06	16.2	*qGW2*	-	-	-	-
	S2_19267209	2	19267209	6.25E-05	15.9	*qGW2*	-	-	-	-
	S2_19251646	2	19251646	9.48E-05	15.8	*qGW2*	-	-	-	-
	S3_16738452	3	16738452	2.20E-07	16.9	*qGW3*	qGL_3	16,729,501	16,735,109	RAP DB
	S3_16790082	3	16790082	6.99E-07	16.7	*qGW3*	qGL_3	16,729,501	16,735,109	RAP DB
	S3_16736739	3	16736739	1.73E-06	16.5	*qGW3*	qGL_3	16,729,501	16,735,109	RAP DB
	S5_5391586	5	5391586	4.30E-08	17.2	*qGW5*	*qGW-5b*	5,915,709	7,810,160	Gramene
	S7_24575488	7	24575488	1.58E-09	17.8	*qGW7*	*grb7-2*	22532352	25188107	qtaro
	S7_24505270	7	24505270	6.17E-08	17.1	*qGW7*	*grb7-2*	22532352	25188107	qtaro
	S7_24518753	7	24518753	1.16E-06	16.6	*qGW7*	*grb7-2*	22532352	25188107	qtaro
	S8_26496216	8	26496216	5.52E-05	15.9	*qGW8*	-	-	-	-
	S8_26177616	8	26177616	6.51E-05	15.9	*qGW8*	-	-	-	-

Supplementary Table S3 Continued: Candidate QTLs and co-located QTLs detected by GWAS for 16 traits using MAGIC global population.

Trait	SNP	Chr	Pos	P.value	PVE (%)	Detected QTLs (Term)	Reported QTLs	Start	End	DB
**GL**	S3_16790082	3	16790082	2.56E-21	18.5	*QGL3*	*qGL_3*	16,729,501	16,735,109	RAP DB
	S3_16738452	3	16738452	1.74E-20	18.1	*QGL3*	*qGL_3*	16,729,501	16,735,109	RAP DB
	S3_16736739	3	16736739	1.18E-19	17.7	*QGL3*	*qGL_3*	16,729,501	16,735,109	RAP DB
	S3_22283454	3	22283454	4.87E-07	12.0	*QGL3.1*	*qGW3*	18350837	22306369	qtaro
	S3_20799507	3	20799507	6.80E-07	11.9	*QGL3.1*	*qGW3*	18350837	22306369	qtaro
	S3_20691702	3	20691702	7.73E-07	11.9	*QGL3.1*	*qGW3*	18350837	22306369	qtaro
	S11_21594187	11	21594187	4.01E-08	12.5	*QGL11*	-	-	-	-
**CHALKY**	S4_22772120	4	22772120	1.51E-05	3.2	*qCLK4*	-	-	-	-
**AC**	S6_1760469	6	1760469	1.30E-25	19.3	*qAC6*	*amy6*	1,764,586	5,425,631	Gramene
	S6_1742193	6	1742193	3.47E-25	19.1	*qAC6*	*amy6*	1,764,586	5,425,631	Gramene
	S6_1892084	6	1892084	1.88E-24	18.7	*qAC6*	*amy6*	1,764,586	5,425,631	Gramene
**Fe**	S3_16789935	3	16789935	1.59E-05	4.8	*qFE3*	-	-	-	-
	S3_16789932	3	16789932	2.09E-05	4.8	*qFE3*	-	-	-	-
	S3_16736681	3	16736681	3.23E-05	4.7	*qFE3*	-	-	-	-
**Zn**	S1_40372091	1	40372091	5.59E-05	17.6	*QZn1*	-	-	-	-
	S5_24312726	5	24312726	5.01E-06	18.0	*QZn5*	*rMQTL5.2*	23,906,571	25,164,524	Jin, T. et al (2015)
	S5_24280680	5	24280680	6.05E-06	17.9	*QZn5*	*rMQTL5.2*	23,906,571	25,164,524	Jin, T. et al (2015)
	S5_24583603	5	24583603	6.26E-06	17.9	*QZn5*	*rMQTL5.2*	23,906,571	25,164,524	Jin, T. et al (2015)
	S7_29281096	7	29281096	9.29E-12	20.1	*QZn7*	*$qZn_{7.1}$*	29,323,098	29,324,607	RAP DB
	S7_29285222	7	29285222	1.42E-10	19.6	*QZn7*	*$qZn_{7.1}$*	29,323,098	29,324,607	RAP DB
	S7_29285320	7	29285320	2.23E-10	19.6	*QZn7*	*$qZn_{7.1}$*	29,323,098	29,324,607	RAP DB
**GYLD**	S3_1222496	3	1222496	1.37E-05	9.2	*qGYLD3*	*qDF_3*	1,270,320	1,300,273	RAP DB
	S3_519745	3	519745	5.80E-05	9.0	*qGYLD3*	*Hd9*	975995	1427051	qtaro
	S3_519765	3	519765	5.80E-05	9.0	*qGYLD3*	*Hd9*	975995	1427051	qtaro
	S4_31340357	4	31340357	8.01E-05	8.9	*qGYLD4*	*qGY4-1*	30,334,770	31,471,486	Gramene
	S4_31340355	4	31340355	8.01E-05	8.9	*qGYLD4*	*qGY4-1*	30,334,770	31,471,486	Gramene
	S4_30601183	4	30601183	8.66E-05	8.9	*qGYLD4*	*qGY4-1*	30,334,770	31,471,486	Gramene

Supplementary Table S4. Candidate QTLs and co-located QTLs identified for 16 traits by interval mapping using MAGIC global population. (Green colours are QTL accession ID in Gramene database)

Trait	Chr	Left Marker	Right Marker	P-value	PVE (%)	Detected QTL	Reported QTLs	Start	End	DB
**PHT**	1	S1_38320382	S1_38366724	0	38.71	*qPHT1*	qPH_1	38,382,385	38,385,469	RAP DB
**PTN**	3	S3_33887305	S3_33947047	2.03E-08	4.68	*qTn3*	*tn3-4*	32,945,649	32,949,568	Gramene
**DTF**	3	S3_1298618	S3_1326339	0	14.11	*qDTF3*	qDF_3	1,270,320	1,300,273	RAP DB
	6	S6_9291056	S6_9317830	1.61E-08	3.89	*qDTF6*	*Hd1*	9,282,505	9,327,178	Gramene
	10	S10_15240779	S10_15308947	6.09E-06	3.16	*qDTF10*	AQCU191	15,800,310	15,802,326	Gramene
	11	S11_26417020	S11_26444568	1.90E-06	2.81	*qDTF11*	*hd11*	25,617,694	25,618,047	Gramene
	12	S12_18903812	S12_18959853	1.67E-07	3.07	*qDTF12*	*QHd12*	19,628,443	19,628,925	Gramene
**PNL**	1	S1_36339891	S1_36388187	8.97E-09	4.99	*qPNL1.2*	*ng1*	34264553	36658883	qtaro
	7	S7_25670580	S7_25740983	1.99E-08	4.82	*qPNL7*	*PL* (AQFW104)	17,525,817	25,775,868	Gramene
**SPAD**	1	S1_38608785	S1_38636497	3.22E-05	2.99	*qCHP1*	*QDg1*	32,987,234	37,889,506	Gramene
**FG**	2	S2_5719097	S2_5755777	1.00E-04	2.37	*qFG2*	AQFW259	5,262,891	6,916,662	Gramene
	8	S8_17190812	S8_17220500	7.90E-05	2.43	*qFG8.1*	*QSf8*	13,927,893	20,650,257	Gramene
**UF**	2	S2_21055606	S2_21301325	4.69E-05	2.9	*qUF2*	-	-	-	-
**TGN**	4	S4_31242011	S4_31306595	3.98E-07	4.29	*qGN4*	*gn-4*	30,630,093	34,698,383	Gramene
	6	S6_6798463	S6_6827955	0.000206	2.78	*qGN6*	*tns6*	6023974	9537572	qtaro
**GWT**	4	S4_28901212	S4_29032959	8.68E-05	3.05	*qGWT4.3*	*gpp4*	21645173	34244441	qtaro
	6	S6_12427481	S6_12462746	0.00029	2.8	*qGWT6*	*gw-6*	9282143	17933378	qtaro
**GW**	3	S3_16789932	S3_16835528	0.000122	4.29	*qGW3*	qGL_3	16,729,501	16,735,109	RAP DB
	5	S5_6073584	S5_6193620	2.51E-08	6.56	*qGW5*	*qGW-5b*	5,915,709	7,810,160	Gramene
	7	S7_25362785	S7_25403593	5.16E-13	8.72	*qGW7*	*grb7-2*	22532352	25188107	qtaro
	9	S9_490750	S9_549225	0.000249	3.79	*qGW9*	-	-	-	-
**GL**	3	S3_16835528	S3_16881568	0	15.38	*QGL3*	qGL_3	16,729,501	16,735,109	RAP DB
	4	S4_24200211	S4_24235414	0.000703	3.86	*QGL4*	-	-	-	-
	4	S4_33153080	S4_33186346	8.92E-06	4.43	*QGL4.1*	AQEO011	31,065,233	34,698,383	Gramene
	7	S7_24637566	S7_24667807	5.76E-07	4.52	*QGL7*	AQEO012	24,085,141	25,990,300	Gramene
**CHALKY**	2	S2_28138470	S2_28176697	6.26E-07	4.28	*qCLK2*	*qER-2b*	27533328	28207849	qtaro
**AC**	1	S1_3573499	S1_3609856	0.000395	2.85	*qAC1*	-	-	-	-
	6	S6_2242943	S6_2343850	0	22.43	*qAC6*	*amy6*	1,764,586	5,425,631	Gramene
	6	S6_28201779	S6_28254038	0.000331	2.72	*qAC6.1*	*ac6.1*	25,691,434	30,447,453	Gramene
**FE**	6	S6_8338324	S6_8367701	7.08E-05	2.81	*qFE6*	-	-	-	-
**ZN**	1	S1_31264631	S1_31502695	2.71E-08	5.33	*QZn1.1*	-	-	-	-
	7	S7_29490504	S7_29519361	2.66E-13	7.71	*QZn7*	$qZn_{7.1}$	29,323,098	29,324,607	RAP DB
**GYLD**	2	S2_5364882	S2_5474837	6.31E-06	2.8	*qGYLD2*	-	-	-	-
	3	S3_1775265	S3_1827398	8.32E-09	4.41	*qGYLD3*	*Hd9*	975995	1427051	qtaro

Supplementary Table S5. Candidate QTLs and co-located QTLs identified for 16 traits by Bayesian networking using MAGIC global population. (Green colours are QTL accession ID in Gramene database)

Trait	Encoded Marker	SNP_Marker	Detected QTLs (Term)	Reported QTLs	Start	End	DB
**PHT**	G973	S1_38286772	*qPHT1*	qPH_1	38,382,385	38,385,469	RAP-DB
	G977	S1_38384548	*qPHT1*	qPH_1	38,382,385	38,385,469	RAP-DB
	G6966	S10_20045466	*qPHT10*	*eui2*	19184232	19184413	qtaro
	G2763	S3_33693235	*qPHT3.1*	*pla3/gp*	33710350	33719780	qtaro
**PTN**	G6490	S9_19628945	*qTn9*	*gy9*	19464779	22021406	qtaro
	G1974	S3_1195479	*qTn3.1*	*Hd9*	975995	1427051	qtaro
	G1566	S2_20894023	*qTn2*	*nt2.1*	20,794,972	20,795,112	Gramene
**DTF**	G1974	S3_1195479	*qDTF3*	qDF_3	1,270,320	1,300,273	RAP-DB
	G6078	S8_26741308	*qDTF8.1*	*QHd8b*	27,822,512	27,825,271	qTaro
	G3745	S5_6696580	*qDTF5*	AQFW190	5,255,880	6,700,408	Gramene
	G5531	S8_3596776	*qDTF8*	*qDTH8*	3170545	5333855	qtaro
**PNL**	G5427	S7_29285320	*qPNL7.1*	*fzp*	28959761	28961248	qtaro
	G977	S1_38384548	*qPNL1.1*	qPH_1	38,382,385	38,385,469	RAP-DB
	G5322	S7_23358106	*qPNL7*	*PL* (AQFW104)	17,525,817	25,775,868	Gramene
	G7243	S11_11196796	*qPNL11*	-	-	-	-
	G5395	S7_27812448	*qPNL7.2*	-	-	-	-
**SPAD**	G973	S1_38286772	*qCHP1*	*QDg1*	32,987,234	37,889,506	Gramene
	G3226	S4_20620638	*qCHP4*	*QDg4a*	18,824,746	20,519,179	Gramene
	G6677	S10_3844218	*qCHP10*	-	-	-	-
	G2611	S3_27416059	*qCHP3*	*QFlw3*	25891087	27899169	Gramene
**FG**	G6263	S9_10480864	*qFG9*	-	-	-	-
**UF**	G1575	S2_21522115	*qUF2*	-	-	-	-
	G4153	S5_28311001	*qUF5.1*	*qpss5.1*	28,850,701	29,193,114	Gramene
	G7891	S12_17575885	*qUF12*	-	-	-	-
	G3749	S5_7024543	*qUF5*	AQDS028	6,132,767	6,133,121	Gramene
	G3214	S4_20319632	*qUF4*	-	-	-	-
**GWT**	G1677	S2_25256653	*qGWT2*	*yld2.1*	21658702	26758298	qtaro
**TGN**	G639	S1_25071517	*qGN1.1*	-	-	-	-
	G3378	S4_27071141	*qGN4.1*	*gpp4*	21645173	34244441	qtaro
**GW**	G5326	S7_23508951	*qGW7*	*grb7-2*	22532352	25188107	qtaro
	G3716	S5_5391586	*qGW5*	*qGW-5b*	5,915,709	7,810,160	Gramene
	G6077	S8_26496216	*qGW8*	-	-	-	-
	G1536	S2_19219429	*qGW2*	-	-	-	-
	G2000	S3_2111982	*qGW3.1*	AQFU044	1,945,837	1,945,949	Gramene
	G1719	S2_26305798	*qGW2.1*	AQDH006	24,565,710	28,690,518	Gramene
	G2607	S3_27161149	*qGW3.2*	AQF146	27,127,661	27,128,592	Gramene
	G3378	S4_27071141	*qGW4*	AQT018	26,857,374	26,857,637	Gramene
**GL**	G2414	S3_16804378	*QGL3*	qGL_3	16,729,501	16,735,109	RAP-DB
	G4280	S6_4849135	*QGL6*	*qLW-6*	4234080	5096867	qtaro

Supplementary Table S5 Continued: Candidate QTLs and co-located QTLs identified for 16 traits by Bayesian networking using MAGIC global population. (Green colours are QTL accession ID in Gramene database)

Trait	Encoded Marker	SNP_Marker	Detected QTLs (Term)	Reported QTLs	Start	End	DB
**CHALKY**	G7537	S11_28940588	*qCLK11*	-	-	-	-
	G6072	S8_26351053	*qCLK8*	qPGWC8	26637711	28166213	qtaro
	G1677	S2_25256653	*qCLK2.1*	-	-	-	-
**AC**	G4247	S6_2395083	*qAC6*	*amy6*	1,764,586	5,425,631	Gramene
	G4232	S6_1594459	*qAC6*	*amy6*	1,764,586	5,425,631	Gramene
	G4240	S6_1875657	*qAC6*	*amy6*	1,764,586	5,425,631	Gramene
	G4231	S6_1577068	*qAC6*	*amy6*	1,764,586	5,425,631	Gramene
	G1558	S2_20416453	*qAC2*	*OsTudor-SN*	19956716	19963395	qtaro
	G5723	S8_11527473	*qAC8*	-	-	-	-
	G2553	S3_23085395	*qAC3*	-	-	-	-
**FE**	G7537	S11_28940588	*qFE11*	-	-	-	-
	G6544	S9_21464008	*qFE9*	-	-	-	-
	G175	S1_5861931	*qFE1*	-	-	-	-
	G3378	S4_27071141	*qFE4*	-	-	-	-
**ZN**	G5427	S7_29285320	*QZn7*	$qZn_{7.1}$ *(OsNAS3)*	29,323,098	29,324,607	RAP-DB
	G2772	S3_34454955	*QZn3*	-	-	-	-
	G1064	S1_40397644	*QZn1*	-	-	-	-
	G4833	S6_28415597	*QZn6*	-	-	-	-
	G4102	S5_24583603	*QZn5*	*rMQTL5.2*	23,906,571	25,164,524	Jin, T. et al (2015)
	G8099	S12_27193387	*QZn12*	-	-	-	-
	G7238	S11_10977733	*QZn11*	-	-	-	-
	G158	S1_5302109	*QZn1.2*	-	-	-	-
**GYLD**	G1969	S3_985889	*qGYLD3*	*Hd9*	975995	1427051	qtaro
	G3247	S4_21869643	*qGYLD4.1*	*gpp4*	21645173	34244441	qtaro
	G4297	S6_5209182	*qGYLD6*	ID210	3459492	6023472	qtaro

Supplementary Table S6. Top candidate genes of significant genomic regions detected by gene association analysis.

**Trait**	**GENE**	**CHR**	**START**	**STOP**	**ZSTAT**	**P**
GYLD_*qDF3*	LOC_Os03g01780	3	485271	486556	3.7927	7.45E-05
	LOC_Os03g03164	3	1340052	1345295	3.7114	0.0001031
	LOC_Os03g01860	3	519135	519795	3.6104	0.0001528
	LOC_Os03g02960	3	1194255	1196505	3.4557	0.0002744
	LOC_Os03g02970	3	1195075	1204839	3.4557	0.0002744
AC_*Amy*	LOC_Os06g04190	6	1754351	1760884	10.395	1.30E-25
	LOC_Os06g04169	6	1736249	1742581	10.301	3.47E-25
	LOC_Os06g04360	6	1856820	1861053	10.011	6.84E-24
	LOC_Os06g04280	6	1816175	1819804	9.6676	2.07E-22
	LOC_Os06g04230	6	1784311	1784827	9.3812	3.26E-21
ZN_*OsFRDL4*	LOC_Os01g69870	1	40371946	40373618	3.4009	0.0003358
	LOC_Os01g69910	1	40397303	40403249	3.3855	0.0003552
ZN_*rMQTL5.2*	LOC_Os05g41450	5	24275818	24280710	4.7738	9.04E-07
	LOC_Os05g41460	5	24279602	24281856	4.5858	2.26E-06
	LOC_Os05g41510	5	24301347	24311126	4.5589	2.57E-06
	LOC_Os05g41660	5	24383180	24384628	4.2627	1.01E-05
	LOC_Os05g41480	5	24287569	24289620	4.0721	2.33E-05
ZN_*qZn7.1*	LOC_Os07g48940	7	29280094	29282008	6.7168	9.29E-12
	LOC_Os07g49110	7	29392447	29401913	6.0933	5.53E-10
	LOC_Os07g49000	7	29338447	29341163	5.6864	6.49E-09
	LOC_Os07g49114	7	29404417	29405293	5.4038	3.26E-08
	LOC_Os07g48970	7	29303615	29309383	5.2248	8.72E-08
