# Supplementary material for: Exploring genetic architecture of grain yield and quality traits in a 16-way indica by japonica rice MAGIC global population
Source: Sci Rep. 2019 Dec 20;9:19605. doi: 10.1038/s41598-019-55357-7 (PMC6925145; doi:10.1038/s41598-019-55357-7)
Supplement: Supplementary file 2 — Supplementary Information [file 41598_2019_55357_MOESM2_ESM.pdf]

Exploring genetic architecture of grain yield and quality traits in a 16-way *indica* by *japonica* rice MAGIC global population

Hein Zaw[1,3], Chitra Raghavan[1], Arnel Pocsedio[1], B.P. Mallikarjuna Swamy[1], Mona Liza Jubay[1], Rakesh Kumar Singh[4], Justine Bonifacio[1], Ramil Mauleon[1], Jose E. Hernandez[2], Merlyn S. Mendioro[2], Glenn B Gregorio[1,2], Hei Leung*[1]

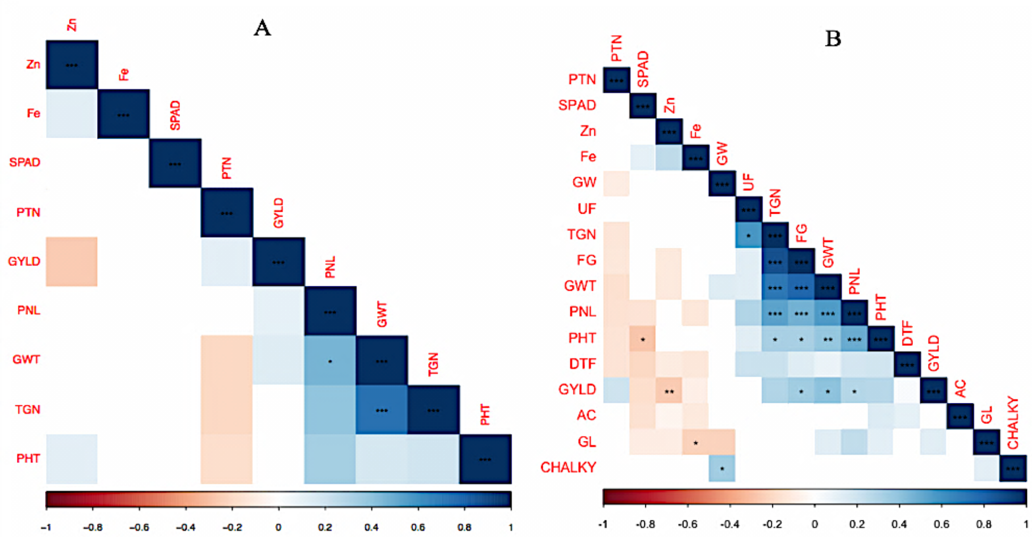

Supplementary Figure S1: (A) Correlations among grain yield, agronomic and biofortification traits during 2015DS, and (B) correlations among grain yield, agronomic, grain quality and biofortification traits during 2016DS. *Significant at $p < 0.05$, **Significant at $p < 0.01$ and ***Significant at $p < 0.001$. PHT, plant height; PTN, number of productive tillers; DTF, days-to-flowering; PNL, panicle length; SPAD, chlorophyll content index; FG, number of filled grains; UF, number of unfilled grains; GWT, grain weight per panicle; TGN, grain number per panicle; GYLD, grain yield; GW, grain width; GL, grain length; CHALKY, chalkiness; AC, amylose content; Zn, grain zinc and Fe, grain iron.

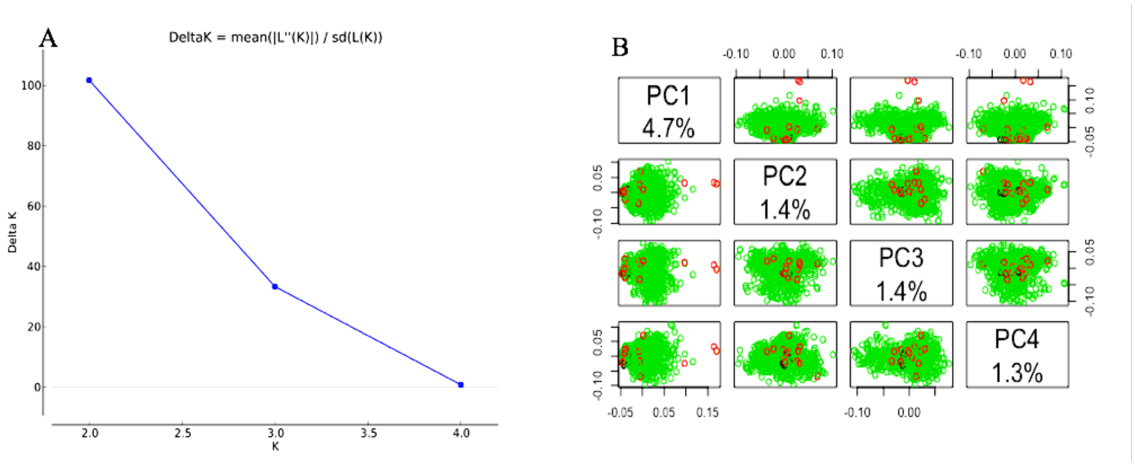

Supplementary Figure S2: (A) Population structure in MAGIC global population (MGP). X-axis shows number of clusters (K) and Y-axis shows ΔK values based on the change rate in log probabilities between two successive K values. The highest ΔK value is 100 at $K = 2$. (B) Principal component analysis (PCA) in MGP. Of 16 parents, two parents were observed in counting the wider variations of the first PC. Red and black circles represent parents and checks, respectively while green circles represent MAGIC RILs.

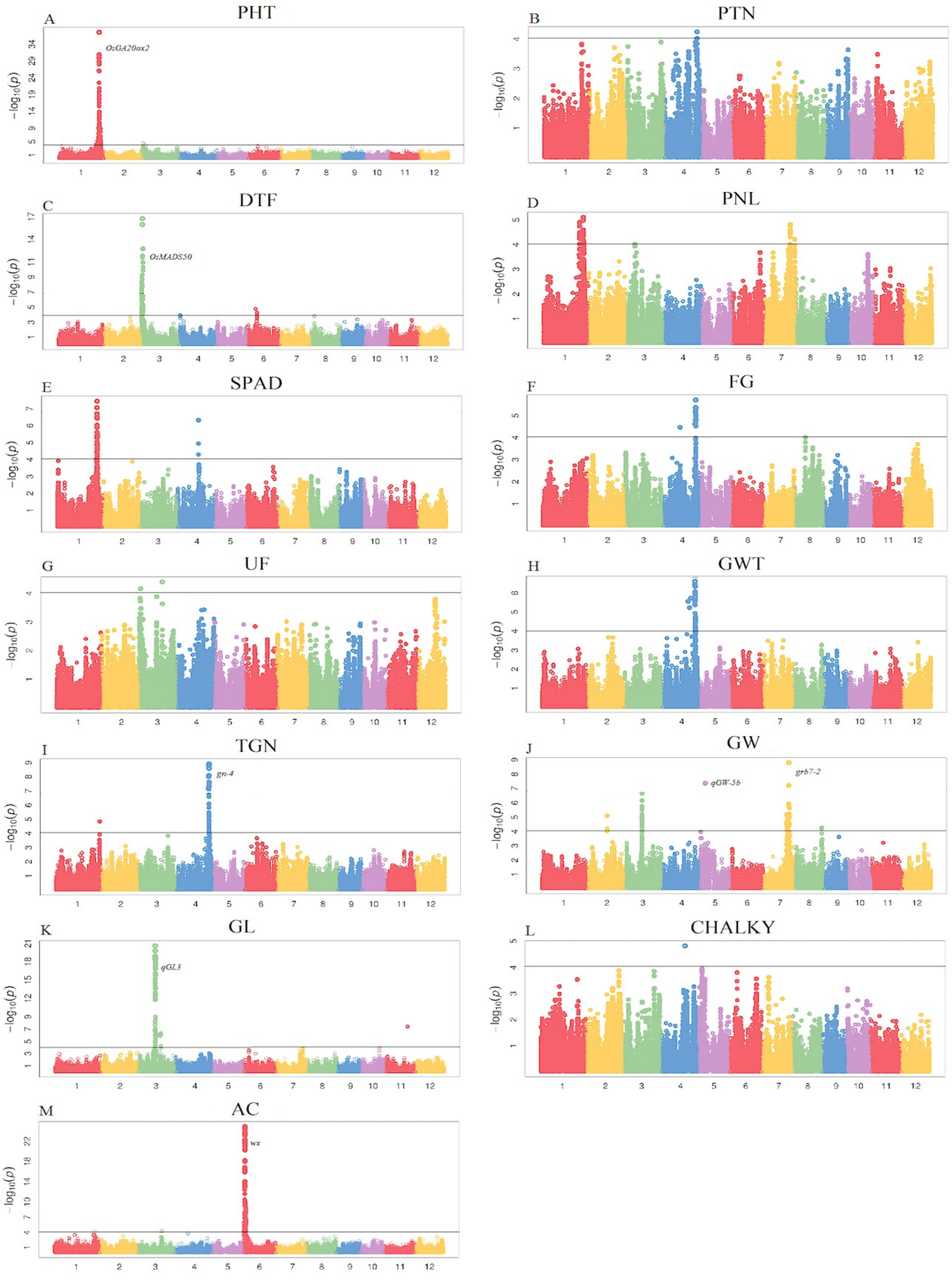

Supplementary Figure S3: Manhattan plots showing associated significant SNP markers for (A) PHT, plant height; (B) PTN, number of productive tillers; (C) DTF, days-to-flowering; (D) PNL, panicle length; (E) SPAD, chlorophyll content index; (F) FG, number of filled grains; (G) UF, number of unfilled grains; (H) GWT, grain weight per panicle; (I) TGN, grain number per panicle; (J) GW, grain width; (K) GL, grain length, (L) CHALKY, chalkiness and (M) AC, amylose content. X-axis shows chromosome number and Y-axis shows $-\log_{10}(p)$. The horizontal line indicates threshold p-value at significant level ($p < 0.0001$).

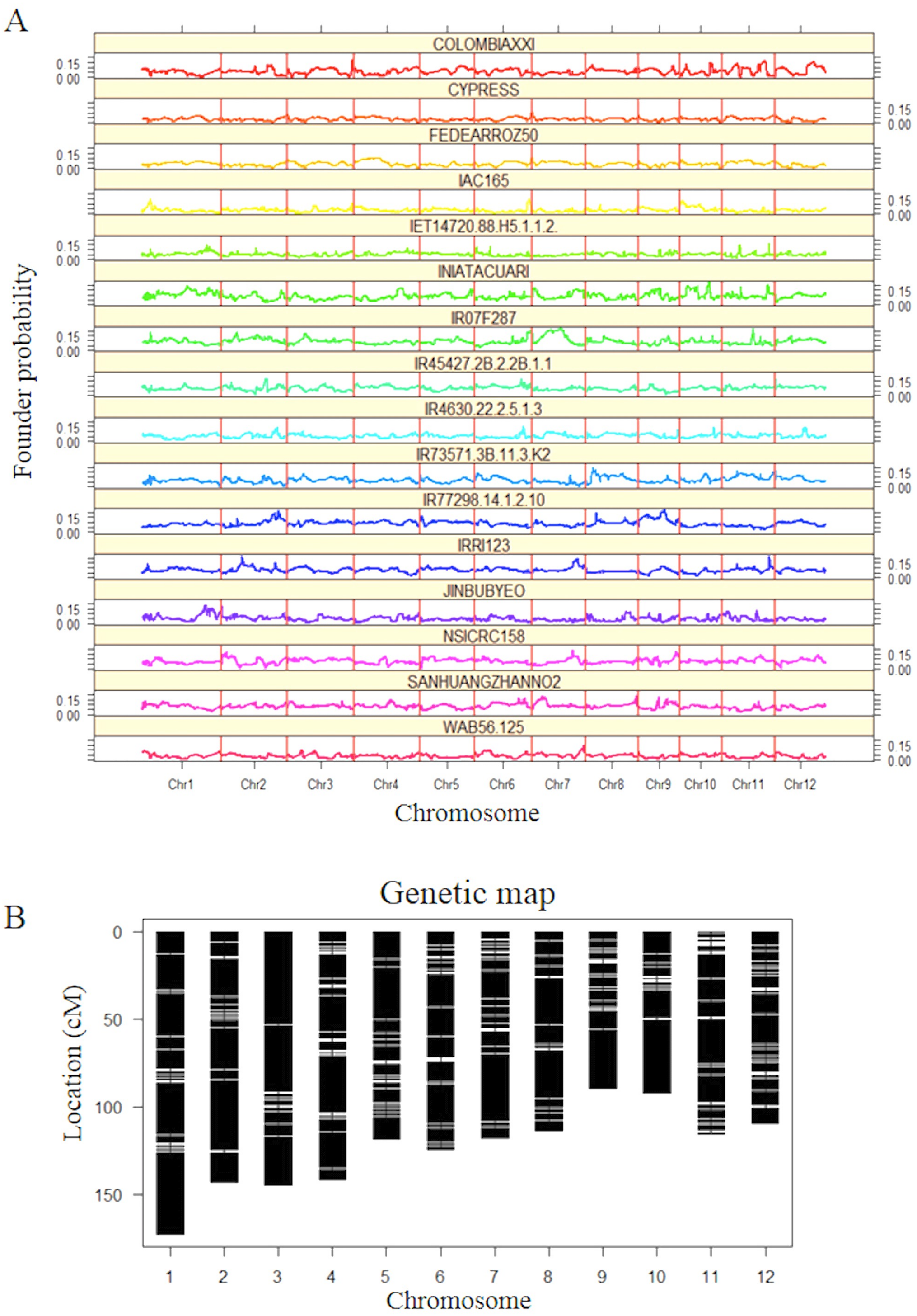

Supplementary Figure S4: (A) Diagram showing probability of 16 founder parents in MAGIC global population. X-axis shows number of chromosomes and Y-axis shows founder probability (B) Genetic map of MAGIC global population. X-axis shows chromosome number and Y-axis shows locations of genetic markers (cM).

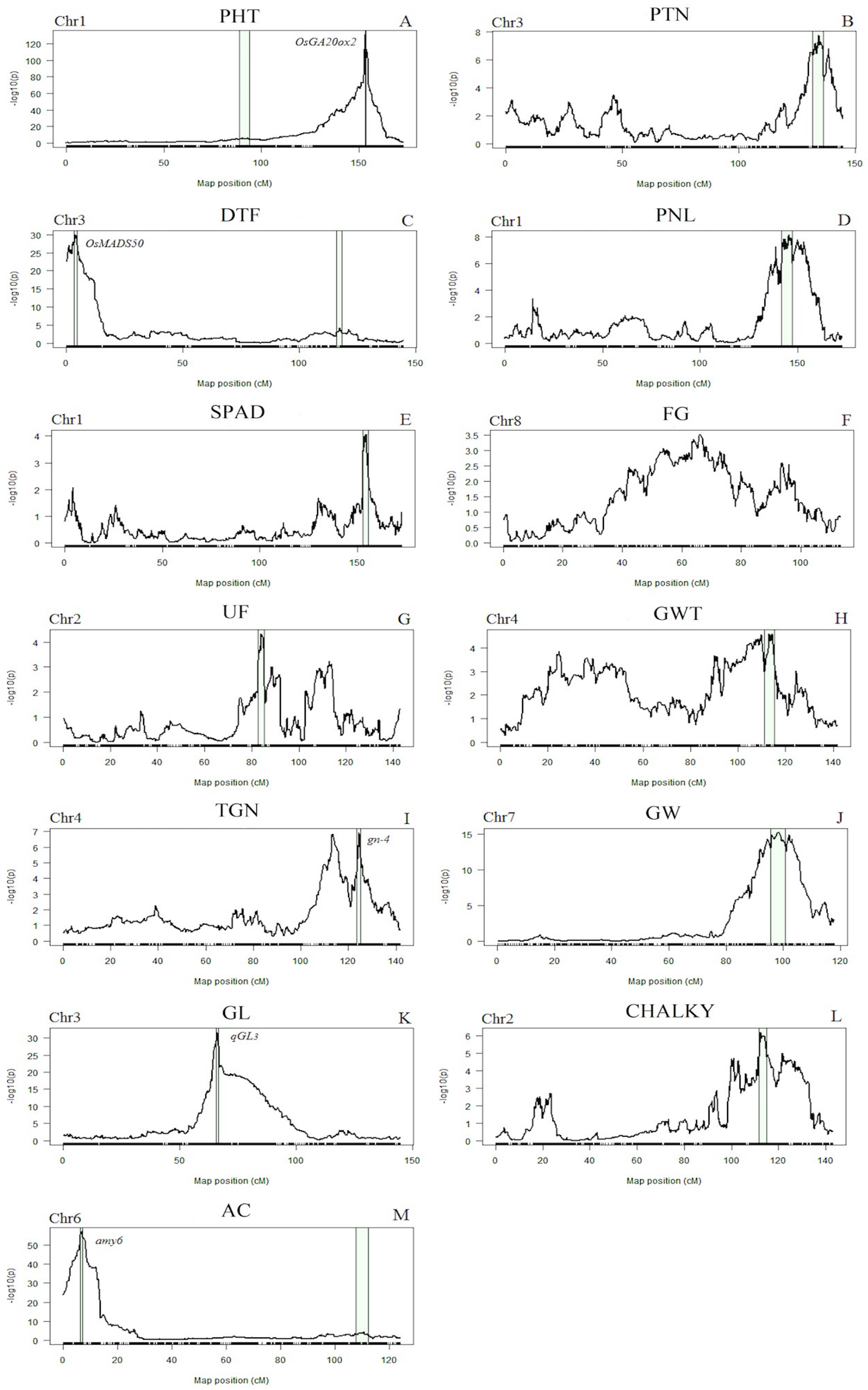

Supplementary Figure S5: Multi-parent interval mapping showing the significant QTLs (A) PHT, plant height; (B) PTN, number of productive tillers; (C) DTF, days-to-flowering; (D) PNL, panicle length; (E) SPAD, chlorophyll content index; (F) FG, number of filled grains; (G) UF, number of unfilled grains; (H) GWT, grain weight per panicle; (I) TGN, grain number per panicle; (J) GW, grain width; (K) GL, grain length, (L) CHALKY, chalkiness and (M) AC, amylose content. Light green colour indicates confident intervals of QTL regions. X-axis shows map position (cM) on chromosomes and Y-axis shows $-\log_{10}(p)$.

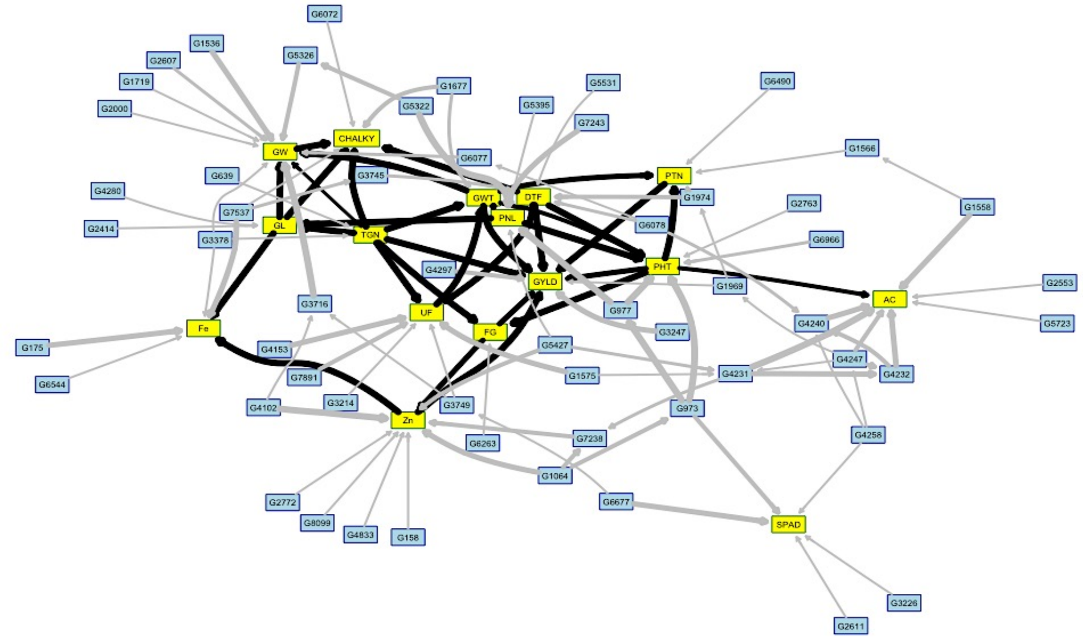

Supplementary Figure S6: Bayesian networking showing trait-trait, trait-marker and marker-marker relationships for 16 measured traits. PHT, plant height; PTN, number of productive tillers; DTF, days-to-flowering; PNL, panicle length; SPAD, chlorophyll content index; GYLD, grain yield; FG, number of filled grains; UF, number of unfilled grains; GWT, grain weight per panicle; TGN, grain number per panicle; GW, grain width; GL, grain length, CHALKY, chalkiness; AC, amylose content; Zn, grain zinc and Fe, grain iron. Light blue and yellow boxes represent encoded SNP markers and traits; black and gray arrows represent the relationships between traits and markers whereas strength of relationships is indicated by thickness of arrows.

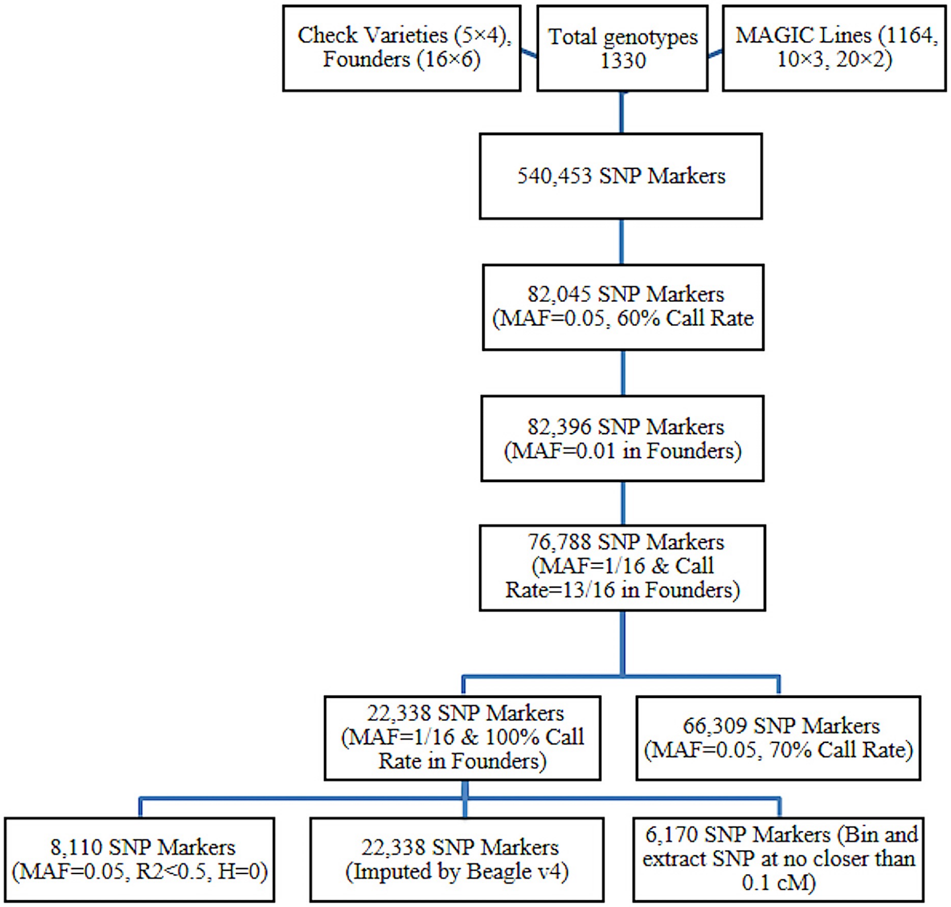

Supplementary Figure S7: Scheme of marker filtering post GBS pipeline to generate different SNP marker sets.
